# Immunosuppressive tumor microenvironment in pancreatic cancer: mechanisms and therapeutic targets

**DOI:** 10.3389/fimmu.2025.1582305

**Published:** 2025-05-15

**Authors:** Da Pan, Xinyue Li, Xiao Qiao, Qiqi Wang

**Affiliations:** ^1^ Department of Gastroenterology, Wenzhou Central Hospital, Wenzhou, China; ^2^ Department of Gastroenterology, The Dingli Clinical College of Wenzhou Medical University, Wenzhou, China; ^3^ First College for Clinical Medicine, Xuzhou Medical University, Jiangsu, Xuzhou, China; ^4^ Department of Gastroenterology, The Affiliated Huaian Hospital of Xuzhou Medical University, Huaian, China

**Keywords:** pancreatic cancer, tumor microenvironment, regulatory T cells, immune suppression, PD-1

## Abstract

Pancreatic cancer is projected to become the second leading cause of cancer−related death by 2030. Conventional interventions including surgery, radiotherapy, and chemotherapy provide only modest survival benefits, underscoring an urgent need for more effective therapies. Although immunotherapy has revolutionized the management of several solid tumors, its clinical benefit in pancreatic cancer has so far been disappointing. Mounting evidence indicates that a highly immunosuppressive tumor microenvironment (TME), dominated by tumor−associated macrophages (TAMs), myeloid−derived suppressor cells (MDSCs), and regulatory T cells (Tregs), drives immune evasion, tumor progression, metastasis, and chemoresistance through complex cytokine and chemokine networks. This review summarizes current knowledge of these immunosuppressive mechanisms and provides emerging strategies aimed at re−educating or depleting these cellular constituents to enhance the efficacy of immunotherapy in pancreatic cancer.

## 1 Introduction

Pancreatic cancer is a highly malignant digestive system tumor with subtle and non-specific clinical symptoms, making early diagnosis difficult. By 2030, it is expected to become the second leading cause of cancer-related deaths globally (1). Traditional treatments like surgery, radiation, and chemotherapy are limited in efficacy (2, 3), contributing to poor prognosis (4, 5). Immunotherapy has shown promise in treating various cancers, but clinical trials for pancreatic cancer have not met expectations. One key challenge is the immunosuppressive tumor microenvironment (TME), which plays a critical role in the tumor’s initiation, development, and prognosis. The TME of pancreatic cancer is characterized by immune cell infiltration (6, 7), primarily of immunosuppressive cells such as pancreatic stellate cells, regulatory T cells (Tregs), myeloid-derived suppressor cells (MDSCs) (8), and tumor-associated macrophages (TAMs) (9). These cells secrete immunosuppressive molecules that inhibit the function of anti-tumor immune cells, promote immune evasion, and enhance tumor progression and metastasis (1, 10, 11). This review summarizes the mechanisms of the immunosuppressive components in the pancreatic cancer TME, aiming to provide insights into its immunotherapy.

## 2 The role of immune cells in the pancreatic cancer TME

Pancreatic cancer evades immunity via MHC I downregulation, inhibiting CD8^+^ T cell activation (12–14). Tumor-specific neoantigens may fail to trigger immune responses due to the TME’s immunosuppressive effects (15, 16). Additionally, TGF-β and IDO secreted by cancer cells further impair immune function (17, 18), with Tregs, MDSCs, and TAMs contributing to immune suppression in early stages (19–21).

### 2.1 The role of TAMs in pancreatic cancer

#### 2.1.1 TAMs promote tumor inflammation and immune evasion

TAMs secrete cytokines and chemokines, such as CCL18, which upregulates VCAM-1 in pancreatic cancer via the CCL18/PITPNM3/NF-κB/VCAM-1 pathway, promoting tumor progression (22). They also release IL-10, IL-12, and CCL13, mediating Th2 responses and suppressing T-cell immunity (23). In mouse models, TAM-produced IL-6 and TNF drive inflammation, while IL-6/STAT3 inhibition reduces inflammatory cell infiltration (24). TAMs activate TLR-6/TLR-2 via Versican, expressing inflammation-related genes (25). CTCF promotes pancreatic cancer progression through FLG-AS1-mediated epigenetic mechanisms and macrophage polarization (26). TAMs facilitate immune evasion by secreting IL-10, inducing T cell apoptosis via CD120a/b (27, 28), altering tumor cell phenotypes, and overexpressing B7-H3 via EGFR/MAPK, inhibiting CD8^+^ T cells (29). Arginase I expression depletes L-arginine, suppressing T cell receptors (30), while the hypoxic, low-glucose TME polarizes macrophages to M2-like phenotypes, further impairing T cell function (31).

#### 2.1.2 TAMs promote tumor metastasis and chemoresistance

Tumor-associated macrophages (TAMs) play multifaceted roles in tumor progression through diverse molecular mechanisms. These immune cells facilitate epithelial-mesenchymal transition (EMT) and enhance metastatic potential via TLR4/IL-10 signaling, while simultaneously inducing matrix metalloproteinases (MMP-2/9) through MIP-3α to promote pancreatic ductal adenocarcinoma (PDAC) invasion (32, 33). Under hypoxic conditions, TAMs activate the PI3Kγ/PTEN pathway and upregulate HIF-1/2α, leading to increased production of pro-angiogenic factors (VEGF, TNF-α, IL-1β) and metastasis-promoting mediators (34–36). TAMs produce EGF and VEGF-A, aiding tumor cell circulation entry. β-catenin-driven TAMs enhance metastasis via OSM/STAT3/LOXL2 (37). The TYROBP-mediated M2 polarization further exacerbates these pro-tumoral effects (38). TAMs express HIF-1α, upregulating VEGF, TNF-α, IL-1β, IL-8, PDGF, bFGF, thymidine phosphorylase, and MMPs, promoting angiogenesis (39). VEGF-A recruits VEGFR2^+^ macrophages, forming TAMs (40). In PDAC, hypoxia increases HIF-1/2 in TAMs, upregulating TGF-β and NRF2 to induce VEGF-A (41). Vasohibin-1 is regulated by TGF-β/BMP signaling between TAMs and tumor cells (42). Notably, TAMs significantly contribute to therapeutic resistance in PDAC through multiple pathways: (1) promoting dense stromal formation and IGF/IGF1R activation (43); (2) enhancing EMT-mediated drug evasion (44); (3) driving gemcitabine resistance via TGF-β1/Gfi-1 signaling, which can be attenuated by simvastatin (45); and (4) fostering immunotherapy resistance through CREB3L1-mediated TAM reprogramming within the tumor microenvironment (46).

### 2.2 The role of tumor-associated neutrophils in pancreatic cancer

#### 2.2.1 TANs regulate tumor immunity in pancreatic cancer

TANs regulate immune responses in PDAC through chemokines and cytokines (47), impairing CD8^+^ T cell infiltration and function. Nectin2^+^ and OLR1^+^ TAN phenotypes are associated with T-cell exhaustion. ER stress regulates TAN protumor activities (48). IL-17-induced neutrophil extracellular traps (NETs) contribute to resistance to immune checkpoint inhibitors (ICIs). CXCR2 and its ligands, such as CXCL5, are crucial for TAN recruitment in pancreatic cancer (49). Immunotargeting neutrophils can restore anti-tumor immunity in pancreatic cancer, improving therapeutic outcomes by addressing immune evasion mechanisms.

#### 2.2.2 TANs regulate pancreatic cancer proliferation and metastasis

TANs in the TME can polarize into N1 and N2 phenotypes. Interferon-β promotes N1 polarization, enhancing anti-tumor immunity, while TGF-β and G-CSF induce N2 polarization, supporting tumor growth (50–52). In pancreatic cancer, TANs secrete a proliferation-inducing ligand (APRIL) (53), which was indicated to plays a role in promoting the progression of pancreatic cancer (54). The interaction of TIMP1 with its receptor CD63 activates the ERK pathway, enhancing NETs formation and tumor proliferation (55). PADI4, a key enzyme driving NET formation, accelerates pancreatic cancer growth. In PADI4 knockout mice, tumor growth is slower, and deoxyribonuclease treatment reduces cancer growth by inhibiting NETs (56).

TME fibrosis and collagen deposition facilitate metastasis, with discoidin domain receptor 1 (DDR1) signaling via NF-κB inducing CXCL5 production, recruiting TANs, and enhancing NET formation (57, 58). NETs trap cancer cells, shield them from immune attack, and induce EMT via PADI4 and elastase translocation, promoting metastasis (59). NETs also enhance liver metastasis by recruiting CAFs and hematopoietic stem cells (60). Consistent with this, dense TAN−derived NET lattices have been visualized within hepatic sinusoids before overt metastatic seeding; these structures trap circulating pancreatic cancer cells, facilitate their extravasation, and attract immunosuppressive macrophages, thereby functionally linking NET formation to both immune suppression and metastatic colonization (61, 62). TANs secrete CCL5, promoting cancer cell migration and CD8^+^ T-cell dysfunction via Nectin2 upregulation (48). Angiogenesis is critical for tumor progression. CXCL5/CXCR2 blockade inhibits tumor growth and angiogenesis via activation of the protein kinase B (Akt), extracellular signal-regulated kinase (ERK) pathways. CXCL8 and CXCL12 synergistically enhance endothelial cell migration and proliferation, with MMP-2 activation further promoting angiogenesis (63). Targeting angiogenesis-related factors is a promising research direction.

#### 2.2.3 TANs influence chemoresistance in pancreatic cancer

TANs contribute to chemoresistance, with growth arrest-specific protein 6 (Gas6) from neutrophils promoting cancer cell regeneration via the Gas6/AXL pathway (64). G-CSF enhances neutrophil recruitment and resistance to anti-VEGF therapy, while MEK inhibition reduces G-CSF production and synergizes with anti-VEGF drugs (65). N2 TANs interfere with antigen-presenting cell (APC) maturation, leading to resistance in CD40-targeted therapies (66). Targeting TANs may improve chemotherapy efficacy in pancreatic cancer. IL-17 induced NETs, which play a key role in the resistance to ICIs in pancreatic cancer (67).

### 2.3 Tumor-infiltrating lymphocytes in pancreatic cancer

TILs, including CD4^+^ Th cells, CD8^+^ CTLs, and Tregs, are pivotal in the TME. High CD8^+^ CTL infiltration correlates with improved survival, as these cells induce tumor cell apoptosis via MHC I-dependent perforin, granzyme, TNF, and IFN-γ release (68, 69), though IL-18 receptor signaling impedes their migration (70). Conversely, Tregs suppress antitumor immunity; lipid synthesis inhibition in Tregs enhances immune responses (71). Moncada et al. (72) linked pancreatic cancer cell states to TME composition, revealing clinical implications. Foxp3, a Treg regulator (73), drives immunosuppression via TGF-β secretion, dendritic cell suppression, and CD8^+^ T-cell inhibition (74–76). Treg levels rise from precancerous lesions to adenocarcinoma, associating with metastasis and advanced staging (77). In tumors, Tregs co-infiltrate with CD4^+^/CD8^+^ T cells but suppress CTL activity, promoting immune evasion (78, 79). Tregs interact with (1) CD20^+^ B cells (worse prognosis), (2) CD3^+^CD56^+^ NKT cells, and (3) CD68^+^CD163^+^ macrophages, influencing immune polarization (80). B regulatory cells (Bregs) are elevated in PDAC and linked to progression (81). PDAC cells and Bregs mutually activate via IL-18, with Bregs expressing PD-L1/IL-35 to suppress CD8^+^ T-cell proliferation and IFN-γ production (81–85). Dual IL-18/PD-L1 blockade reduces tumor growth in models, highlighting Breg-cancer crosstalk as a therapeutic target (81).

### 2.4 MDSCs in pancreatic cancer

Myeloid-derived suppressor cells (MDSCs) represent a heterogeneous population of immature myeloid cells that play a critical role in tumor-mediated immune suppression. Although scarcely present in normal pancreatic tissue, these cells accumulate significantly in pathological conditions including pancreatic intraepithelial neoplasia and chronic pancreatitis (86, 87). In advanced pancreatic cancer, tumor-derived factors and inflammatory mediators promote MDSC recruitment and activation, leading to their substantial expansion in the bone marrow, peripheral circulation, and tumor microenvironment (88–90). The immunosuppressive functions of MDSCs are mediated through multiple interconnected mechanisms. These cells generate reactive oxygen species (ROS) in response to cytokines such as TGF-β, IL-10, and IL-6, creating an oxidative environment that impairs immune cell function within the TME. Furthermore, MDSCs express high levels of arginase and nitric oxide synthase, which deplete essential amino acids and disrupt critical signaling pathways including JAK3 and STAT5, ultimately leading to T cell dysfunction and apoptosis. The production of peroxynitrite by MDSCs causes nitration of T cell receptor (TCR) and CD8 molecules, thereby compromising antigen recognition capacity (91).

MDSCs also promote immune tolerance through indirect mechanisms. Under IFN-γ stimulation, they secrete IL-10 and TGF-β to drive regulatory T cell (Treg) differentiation (92) Additionally, MDSCs upregulate PD-L1 expression to directly inhibit T cell activity while simultaneously reducing L-selectin expression, which impairs T cell homing and activation (91). Preclinical studies demonstrate that MDSC depletion in pancreatic cancer models enhances T cell infiltration, suppresses tumor progression, and improves survival outcomes, highlighting these cells as promising therapeutic targets (88). Besides, KRAS mutations occur in 90% of PDAC cases (93), driving tumorigenesis and progression (94). These alterations shape an immunosuppressive TME by expanding MDSCs and depleting dendritic cells, undermining antitumor immunity (95). Combining KRAS inhibition with I/O therapies may thus overcome resistance.

### 2.5 Inflammatory mediators in pancreatic cancer

**Figure 1:**
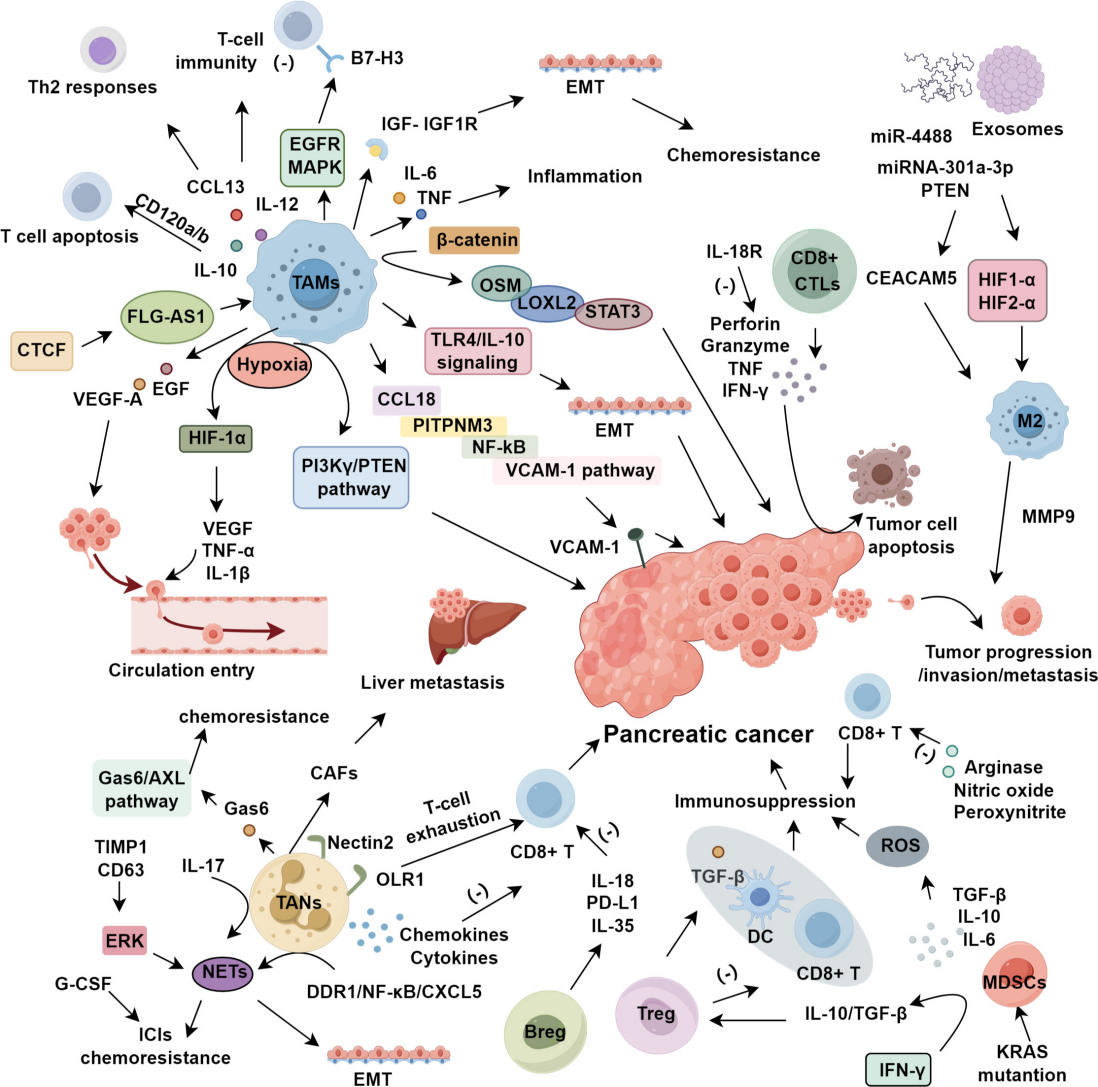
The role of immunosuppressive cells in pancreatic cancer.

The TME is integral to tumorigenesis, development, and metastasis, comprising cancer cells, stromal cells, cytokines, and inflammatory mediators (96–98). Inflammation is closely linked to cancer, with conditions like obesity and diabetes being risk factors for PDAC, supported by the concept of “parainflammation” (99). Tumor cells secrete chemokines to recruit inflammatory cells, as demonstrated by Makoto Sano et al. (100), showing that the CXCR2-CXCL5 axis accelerates invasion and migration in PDAC. CXCR2-dependent regulation modulates the formation, angiogenesis, and metastasis of pancreatic cancer, with CXCR2 also promoting immune evasion and cancer development through autocrine effects, while recent experiments show that cell-autonomous CXCL5 maintains tolerance loops and stromal inflammation by inducing TNF derived from neutrophils in cancer cells (101, 102). Cytokines such as TGF-β, IL-6, IL-10, and TNF-α play critical roles in tumor progression and metastasis. TGF-β enhances invasion via EMT, IL-6 activates JAK2-STAT3 to promote angiogenesis, and TNF-α induces metastasis by activating fibroblasts and promoting VEGF production in PDAC (99) (**Figure 1**).

### 2.6 Exosomes in pancreatic cancer

Exosomes secreted by both eukaryotic and prokaryotic cells contain RNA, DNA, proteins, lipids, and sugars (103–105). In pancreatic cancer, exosomes transfer signaling molecules that promote tumor proliferation, differentiation, and metastasis (106). For example, miR-3960 inhibits TFAP2A in exosomes, counteracting their promotive effects (107). Exosomes, enriched with miRNAs like miR-10b, miR-550, and miR-1246, serve as biomarkers for early diagnosis (108). Exosomes also deliver siRNA targeting oncogenes, like KrasG12D, more effectively than liposomes (109). Exosomes, linked to tumor metastasis, are secreted in the hypoxic microenvironment and enriched with miRNA-301a-3p, phosphatases, and the angiotensin II/PI3Kγ signaling pathway, which induce HIF1-α or HIF2-α to stimulate M2 macrophage polarization, thereby promoting tumor cell metastasis (34, 110). Exosomes derived from hypoxic pancreatic neuroendocrine tumors (PNETs) contain CEACAM5, which facilitates the polarization of TAMs towards the M2 phenotype, thereby enhancing tumor metastasis through the activation of MMP9 (111). Additionally, hypoxic tumor-derived exosomal miR-4488 induces M2 polarization in macrophages, promoting liver metastasis of pancreatic neuroendocrine tumors via the RTN3/FABP5 axis, which drives fatty acid oxidation (112). Exosomes can also influence metastasis and invasiveness via signaling pathways like circ-PDE8A (113). Thus, exosomes have significant clinical value, warranting further exploration in pancreatic and other cancers.

## 3 Therapies targeting immune suppressive cells

### 3.1 Targeting TAMs in treatment

Tumor aggressiveness is influenced by TAM location and quantity. Inhibiting the CCL2/CCR2 axis reduces TAMs TAM recruitment, promotes M1 polarization, and suppresses M2 phenotypes (114, 115). Clinical evidence highlights carlumab (anti-CCL2) for pancreatic cancer (116) and PF-04136309 (CCR2 antagonist) in preclinical/clinical settings (117, 118). CCR2 or CSF-1R targeting augments chemotherapy, curbs metastasis, and amplifies T-cell activity (119). Ultrasound-mediated CSF1/CSF1R blockade depletes macrophages, showing therapeutic potential (120). Clodronate liposomes deplete TAMs, increasing IFN-γ^+^ CD8^+^ T-cell infiltration in PDAC (121). Macrophage repolarization (M2→M1) via LPS, IFN-γ, TLR4 agonists, or paclitaxel/Nab-paclitaxel enhances immunity (122–124). NF-κB inhibition reprograms TAMs, potentially through IFN-γ/CCL2, improving anti-tumor responses (125). CD40 agonists with gemcitabine remodel PDAC’s immune landscape, activating T cells (126). Histamine-rich glycoproteins induce M1 polarization, normalize vasculature, and restore CD8^+^ T-cell function via PI3Kγ inhibition (126, 127). IL-27, produced by activated macrophages, activates JAK-STAT, shifting TAMs from M2 to M1, inhibiting tumor growth, and enhancing gemcitabine efficacy (128). Natural molecules like sphingosine (129) and Urolithin A (130) also modulate M2 polarization in PDAC. Targeting PI3Kγ inhibitors offer new therapies, which enhances macrophage efferocytosis in pancreatic cancer, supporting tumor control when combined with radiotherapy (131). Besides, the phase Ib/II study NCT03767582, which tests the dual CCR2/CCR5 antagonist BMS−813160 together with nivolumab (± GVAX) in locally−advanced PDAC show enhanced intratumoral CD8^+^CD137^+^ T-cells and manageable toxicity, while preclinical data confirmed CCR2 blockade synergizes with anti-PD-1 by increasing CD8^+^ T-cell infiltration and reducing T-regs in PDAC (132).

### 3.2 Targeting TANs in treatment

Current immune therapies targeting TANs focus on inhibiting TAN recruitment via cytokine/chemokine axes. CXCR2 deficiency reduces pancreatic cancer vascular density (101), while blocking CXCR2-CXCL8 enhances PD-1 efficacy (133). Lorlatinib inhibits G-CSF, reduces TAN recruitment, and boosts CD8^+^ T/NK cell cytotoxicity (134). HMGB1 from NETs promotes malignancy, despite thrombomodulin degrades HMGB1 (135). Neutralizing IL-1β inhibits EGFR/ERK activation and EMT (136), while NET targeting mitigates hypercoagulability and thrombosis (137). Inhibiting specific TAN phenotypes: P2RX1-negative neutrophils in liver metastases correlate with PD-L1 expression; their inhibition activates CD8^+^ T cell anti-tumor immunity, suppressing pancreatic cancer progression.

### 3.3 Adoptive TILs cell therapy

Since the 1980s, TILs adoptive cell therapy has evolved, involving extraction, *in vitro* culture, and reinfusion into patients. Rosenberg et al. (138) reported 34%-56% efficacy in melanoma, while its potential in pancreatic cancer remains unexplored but promising. Sakellariou-Thompson et al. (139) demonstrated that CD8^+^ TILs from pancreatic cancer can grow with 4-1BB agonists, supporting clinical feasibility. Targeting immunosuppressive cells, such as CCR4^+^ Tregs in melanoma, shows promise, with CCR4 antibodies depleting Tregs *in vivo* and *in vitro* (140). Mogamulizumab, an anti-CCR4 antibody, is in clinical trials. Bacterial therapy, like Salmonella A1-R, enhances CD8^+^ TILs in pancreatic cancer models, suggesting anti-tumor immunity activation (141, 142). Chemotherapy combined with innate immune agonists improves T cell priming for ICIs (143) (144–146). TNFR2 blockade in PDAC targets Tregs, reducing immunosuppression and T cell exhaustion (147). eIF4G1 overexpression in PDAC correlates with poor prognosis; its inhibition reduces pro-tumor cytokines, promotes M2-TAM polarization (148), and enhances CD8^+^ T cell recruitment (149–151), offering a therapeutic strategy (152).

### 3.4 Anti-CTLA-4/PD-1 therapy

Anti-CTLA-4 and anti-PD-1 antibodies are ICIs that activate CD8^+^ T cell responses. CTLA-4, identified as a T cell checkpoint factor by KRUMMEL et al. (153), but ICIs show limited efficacy in pancreatic cancer due to resistance (154). PD-1 treatment’s effectiveness remains controversial, though PDL-1 overexpression may predict response. Combined anti-PD-L1 and anti-CTLA-4 therapy showed promise in pancreatic neuroendocrine tumors (154). Deng et al. (155) found glucocorticoid receptor (GR) inhibition downregulates PD-L1 and upregulates MHC-I, enhancing immune therapy sensitivity. Other immunosuppressive pathways (TIM3, TIGIT, LAG3, VISTA, CD73) are highly expressed in PDAC (156), suggesting potential therapeutic targets. Oncolytic virotherapy (OVs) using modified viruses (e.g., adenoviruses, herpes simplex viruses) combined with ICIs shows promise, particularly in head and neck squamous cell carcinoma (157). Hyperthermia, as a sensitizer, enhances immune activation and, when combined with gemcitabine, reduces invasion and metastasis in pancreatic cancer cells by promoting apoptosis via reactive oxygen species (158).

### 3.5 Other immune suppressive cells targeted therapies

Exosome-based dual delivery systems, such as iEXO-OXA, enhance immune responses in orthotopic PDAC mice by inducing immunogenic cell death (ICD) and reversing immune suppression. These exosomes improve drug accumulation in tumors while minimizing systemic distribution, promoting innate and adaptive anti-PDAC immunity by enhancing ICD, dendritic cell maturation, and cytotoxic T lymphocyte infiltration (159). GVAX, a GM-CSF gene-transfected pancreatic tumor cell vaccine, combined with low-dose cyclophosphamide, induces anti-tumor immunity, including Treg depletion and tertiary lymphoid structure formation, improving immune cell infiltration in the TME. The combination with nivolumab and urelumab shows promising efficacy in resectable pancreatic cancer (160–162).

## 4 Conclusion

Pancreatic cancer remains a formidable challenge due to its immunosuppressive TME, which facilitates tumor progression, metastasis, and resistance to conventional therapies. The intricate interplay between immune cells, stromal components, and inflammatory mediators creates a hostile environment that limits the efficacy of current treatments. Immunosuppressive cells, as key players in this process, promote immune evasion, angiogenesis, and chemoresistance through diverse mechanisms, including cytokine secretion, NET formation, and stromal remodeling. Novel treatment approaches, including TAM/TAN-directed interventions, adoptive transfer of tumor-infiltrating lymphocytes, blockade of immune checkpoints, and engineered exosome platforms, present viable solutions to circumvent these limitations. However, the complexity of the TME necessitates a multifaceted approach, combining these therapies with conventional treatments to enhance anti-tumor immunity and improve patient outcomes. Future research should focus on elucidating the molecular mechanisms underlying immune suppression in pancreatic cancer and developing innovative, targeted therapies to reprogram the TME and restore effective immune surveillance.
